# *FGFR2* fusion/rearrangement is associated with favorable prognosis and immunoactivation in patients with intrahepatic cholangiocarcinoma

**DOI:** 10.1093/oncolo/oyae170

**Published:** 2024-07-10

**Authors:** Shaoqing Liu, Jialei Weng, Manqing Cao, Qiang Zhou, Min Xu, Wenxin Xu, Zhiqiu Hu, Minghao Xu, Qiongzhu Dong, Xia Sheng, Chenhao Zhou, Ning Ren

**Affiliations:** Department of Liver Surgery and Transplantation, Liver Cancer Institute, Zhongshan Hospital, Fudan University, Key Laboratory of Carcinogenesis and Cancer Invasion, Ministry of Education, Shanghai, 200032, People’s Republic of China; Key Laboratory of Whole-Period Monitoring and Precise Intervention of Digestive Cancer of Shanghai Municipal Health Commission, Shanghai, 201199, People’s Republic of China; Department of Liver Surgery and Transplantation, Liver Cancer Institute, Zhongshan Hospital, Fudan University, Key Laboratory of Carcinogenesis and Cancer Invasion, Ministry of Education, Shanghai, 200032, People’s Republic of China; Key Laboratory of Whole-Period Monitoring and Precise Intervention of Digestive Cancer of Shanghai Municipal Health Commission, Shanghai, 201199, People’s Republic of China; Tianjin Medical University Cancer Institute and Hospital, National Clinical Research Center for Cancer, Tianjin’s Clinical Research Center for Cancer, Key Laboratory of Cancer Prevention and Therapy, Tianjin, 300060, People’s Republic of China; Department of Liver Surgery and Transplantation, Liver Cancer Institute, Zhongshan Hospital, Fudan University, Key Laboratory of Carcinogenesis and Cancer Invasion, Ministry of Education, Shanghai, 200032, People’s Republic of China; Key Laboratory of Whole-Period Monitoring and Precise Intervention of Digestive Cancer of Shanghai Municipal Health Commission, Shanghai, 201199, People’s Republic of China; Department of Liver Surgery and Transplantation, Liver Cancer Institute, Zhongshan Hospital, Fudan University, Key Laboratory of Carcinogenesis and Cancer Invasion, Ministry of Education, Shanghai, 200032, People’s Republic of China; Key Laboratory of Whole-Period Monitoring and Precise Intervention of Digestive Cancer of Shanghai Municipal Health Commission, Shanghai, 201199, People’s Republic of China; Department of Liver Surgery and Transplantation, Liver Cancer Institute, Zhongshan Hospital, Fudan University, Key Laboratory of Carcinogenesis and Cancer Invasion, Ministry of Education, Shanghai, 200032, People’s Republic of China; Key Laboratory of Whole-Period Monitoring and Precise Intervention of Digestive Cancer of Shanghai Municipal Health Commission, Shanghai, 201199, People’s Republic of China; Key Laboratory of Whole-Period Monitoring and Precise Intervention of Digestive Cancer of Shanghai Municipal Health Commission, Shanghai, 201199, People’s Republic of China; Institute of Fudan-Minhang Academic Health System, Minhang Hospital, Fudan University, Shanghai, 201199, People’s Republic of China; Department of Liver Surgery and Transplantation, Liver Cancer Institute, Zhongshan Hospital, Fudan University, Key Laboratory of Carcinogenesis and Cancer Invasion, Ministry of Education, Shanghai, 200032, People’s Republic of China; Key Laboratory of Whole-Period Monitoring and Precise Intervention of Digestive Cancer of Shanghai Municipal Health Commission, Shanghai, 201199, People’s Republic of China; Key Laboratory of Whole-Period Monitoring and Precise Intervention of Digestive Cancer of Shanghai Municipal Health Commission, Shanghai, 201199, People’s Republic of China; Institute of Fudan-Minhang Academic Health System, Minhang Hospital, Fudan University, Shanghai, 201199, People’s Republic of China; Key Laboratory of Whole-Period Monitoring and Precise Intervention of Digestive Cancer of Shanghai Municipal Health Commission, Shanghai, 201199, People’s Republic of China; Institute of Fudan-Minhang Academic Health System, Minhang Hospital, Fudan University, Shanghai, 201199, People’s Republic of China; Department of Pathology, Minhang Hospital, Fudan University, Shanghai, 201199, People’s Republic of China; Department of Liver Surgery and Transplantation, Liver Cancer Institute, Zhongshan Hospital, Fudan University, Key Laboratory of Carcinogenesis and Cancer Invasion, Ministry of Education, Shanghai, 200032, People’s Republic of China; Key Laboratory of Whole-Period Monitoring and Precise Intervention of Digestive Cancer of Shanghai Municipal Health Commission, Shanghai, 201199, People’s Republic of China; Department of Liver Surgery and Transplantation, Liver Cancer Institute, Zhongshan Hospital, Fudan University, Key Laboratory of Carcinogenesis and Cancer Invasion, Ministry of Education, Shanghai, 200032, People’s Republic of China; Key Laboratory of Whole-Period Monitoring and Precise Intervention of Digestive Cancer of Shanghai Municipal Health Commission, Shanghai, 201199, People’s Republic of China; Institute of Fudan-Minhang Academic Health System, Minhang Hospital, Fudan University, Shanghai, 201199, People’s Republic of China

**Keywords:** FGFR2, fusion/rearrangement, prognosis, immune contexture, intrahepatic cholangiocarcinoma

## Abstract

Increasing evidence highlights that fibroblast growth factor receptor 2 (*FGFR2*) fusion/rearrangement shows important therapeutic value for patients with intrahepatic cholangiocarcinoma (ICC). This study aims to explore the association of *FGFR2* status with the prognosis and immune cell infiltration profiles of patients with ICC. A total of 226 ICC tissue samples from patients who received surgery at the Department of Liver Surgery at Zhongshan Hospital, Fudan University, were collected retrospectively and assigned to a primary cohort (*n* = 152) and validation cohort (*n* = 74) group. Fluorescence in situ hybridization was performed to determine *FGFR2* status. Multiplex immunofluorescence (mIF) staining and immunohistochemistry were performed to identify immune cells. Thirty-two (14.2%) ICC tissues presented with *FGFR2* fusion/rearrangement. *FGFR2* fusion/rearrangement was associated with low levels of carcinoembryonic antigen (CEA, *P* = .026) and gamma glutamyl transferase (γ-GGT, *P* = .003), low TNM (*P* = .012), CNLC (*P* = .008) staging as well as low tumor cell differentiation (*P* = .016). Multivariate COX regression analyses revealed that *FGFR2* fusion/rearrangement was an independent protective factor for both overall survival (OS) and relapse-free survival in patients with ICC. Furthermore, correlation analysis revealed that an *FGFR2* fusion/rearrangement was associated with low levels of Tregs and N2 neutrophils and high levels of N1 neutrophils infiltrating into tumors but not with CD8^+^ T-cell or macrophage tumor infiltration. *FGFR2* fusion/rearrangement may exert a profound impact on the prognosis of ICC patients and reprogram the tumor microenvironment to be an immune-activated state. *FGFR2* status may be used for ICC prognostic stratification and as an immunotherapeutic target in patients with ICC.

## Teaser

This study explored the association of FGFR2 status with the prognosis and immune cell infiltration profiles of patients with intrahepatic cholangiocarcinoma.

**Implications for Practice**

ICC is a malignant tumor with high aggressiveness and high mortality. The discovery of new indicators with predictive prognostic value is of great clinical importance for patients with ICC. Fibroblast growth factor receptor 2 (*FGFR2*) fusion/rearrangement in cholangiocarcinoma cells are almost exclusively ICC events, with an incidence of approximately 10%-16%. Our results showed that *FGFR2* alterations can be used as prognostic predictors and are associated with immune activation in ICC.

## Introduction

Intrahepatic cholangiocarcinoma (ICC), a unique bile duct cancer, is a malignant tumor with high aggressiveness and high mortality.^1^ The incidence of ICC and the mortality rate are increasing globally, and it is now the second most common type of primary liver cancer, accounting for approximately 5%-20% of all cases.^2-4^ Most ICCs are detected at advanced stages in clinical practice, mainly because of their insidious development and a lack of effective detection indicators.^5^ Radical surgical resection and postoperative systemic therapy are common treatments for ICC.^6^ Nevertheless, patients with ICC face challenges such as a high risk of recurrence and poor prognosis. Therefore, the discovery of new indicators with predictive prognostic value is of great clinical importance for patients with ICC.

Next-generation sequencing-based genome analysis is used to detect genomic alterations such as mutations, fusions/rearrangements and copy number amplification.^7^ Fibroblast growth factor receptors (FGFRs) are members of the receptor tyrosine kinase family, which mainly includes 4 members, FGFR1-4. Under physiological conditions, FGFRs bind to fibroblast growth factors and undergo homodimerization which induces their autophosphorylation and activates downstream signaling pathways.^8^ Previous genomic analyses have shown that *FGFR2* fusion/rearrangement in cholangiocarcinoma cells are almost exclusively ICC events, with an incidence of approximately 10%-16%.^9^*FGFR2* fusion/rearrangement were initially considered to be specific clinical targets of ICC.^10^ For example, the FIGHT202 trial revealed that drugs targeting *FGFR2* fusion/rearrangement led to objective responses in 35.5% patients with locally advanced or metastatic cholangiocarcinoma.^10^ Pemazyre (pemigatinib) and Lytgobi (Futibatinib) was approved by the US FDA as the drug targeted to cholangiocarcinoma. However, it remains unclear whether *FGFR2* fusion or rearrangement can be used as a predictor of clinical prognosis.

The altered immune profile of tumor tissues is an important feature of malignancies, and immunotherapy has changed tumor treatment strategies in recent decades.^11^ Single-cell RNA sequencing (scRNA-seq) analysis has revealed significant tumor heterogeneity and a high immunosuppressive profile of tumor-infiltrating regulatory T cells (Tregs) in the context of ICC.^12^ ICC phenotype are described according to the tumor heterogeneity and tumor microenvironment, and among these phenotypes, the inflammatory ICC subtype is treated with immune checkpoint blockers.^13^ However, whether *FGFR2* status affects tumor or immune cell function in patients with ICC is still unclear. Exploring the correlation between *FGFR2* status and immune cell infiltration is important for understanding the microenvironment of ICC cells with *FGFR2* mutations.

Herein, tumor specimens from 226 ICC patients were collected, and fluorescence in situ hybridization (FISH) and multiplex immunofluorescence (mIF) were performed to understand the immune landscape of ICC cells with different *FGFR2* statuses. Our results showed that *FGFR2* alterations can be used as prognostic predictors and are associated with immune activation in ICC.

## Materials and methods

### Patient selection

This study retrospectively collected samples from 226 ICC patients who visited the Department of Liver Surgery, Zhongshan Hospital, Fudan University between January 2012 and April 2015. Patients were randomly assigned to a primary cohort (*n* = 152) and validation cohort (*n* = 74) at a 2:1 ratio. The following inclusion criteria were used: (1) initial presentation of ICC with no distant metastasis; (2) diagnosis of ICC based on postsurgical pathological analysis; and (3) complete R0 resection and systemic postoperative treatment. The exclusion criteria were (1) incomplete clinical data and (2) missing follow-up information. All patients received homogeneous means of treatment postoperatively and FGFR2-positive patients did not take FGFR inhibitors. Patients received followed up based on an outpatient registry system, and recurrence-free survival (RFS) and overall survival (OS) were recorded. RFS was defined as the time from surgical removal of the tumor to the first appearance of local or distant metastases. OS was defined as the time from surgical removal of the tumor to death from any cause. This study was approved by the Ethics Committee of Zhongshan Hospital, Fudan University.

### Fluorescence in situ hybridization (FISH)

To detect *FGFR2* rearrangements in the samples, we performed FISH on formalin-fixed paraffin-embedded tumor samples using *FGFR2* (10q26) gene break-apart probe reagent, which marked the gaps between the translocated sequences with red and green (F.01197-01, LBP, China). The samples were first serially sliced into sections with a thickness of 0.2 μm. The sections were placed in an incubator set at 50 °C for 2-3 hours. After removal from the incubator, the sections were denatured by immersion in a denaturing solution consisting of 75% formamide and 2× saline sodium citrate (SSC) and incubation at 75 °C for 3 minutes. The sections were then immediately dehydrated through sequential treatment with 70%, 90%, and 100% ice-cold ethanol for 5 minutes each time and then air-dried. The probes were denatured in a water bath constantly maintained at 75 °C for 5 minutes and then immediately placed in an ice bath (0 °C) for 5 minutes. A 10-μL drop of denatured probe liquid was placed onto the denatured and dehydrated sections, sealed, placed in a humid dark box, and incubated overnight at 37 °C. The next day, the specimens were removed and washed with 50% formamide and 2× SSC at 45 °C for 15 minutes/3 times, followed by a wash with 1× SSC at 45 °C for 15 minutes/3 times. The specimens were lightly washed with 2× SSC at room temperature, and the sections were removed and air-dried. DAPI counterstain was added dropwise to the sections, which were then sealed. Hybridization signal scoring of the sections was performed using a fluorescence microscope (Olympus, Japan) at 10 × 100 high magnification, and 3 fields of view of each section were analyzed. The presence of separate red and/or separate green spectra in more than 20% of the nuclei in a tumor region was considered to indicate a positive FISH result.^14^ The FISH results were confirmed by 2 blinded pathologists.

### Immunohistochemistry staining and multiplexed immunofluorescence staining

Immunohistochemistry (IHC) staining was performed with tissue microarray (TMA) specimens. We used an anti-Foxp3 monoclonal antibody, PD-L1 and CSF1R antibody for IHC staining as previously described.^15^

mIF staining was performed with the TMA specimens. We used anti-myeloperoxidase (MPO), anti-CD11b, anti-CD206, anti-CD80, and anti-CD8 monoclonal antibodies as the primary antibodies, where were incubated with the sections at 4 °C overnight. Next, the specimens were incubated with CY3 (B0059), CY5 (B0060), and FITC (B0061) for 10 minutes. Finally, the specimens were incubated with DAPI (B0025) for 10 minutes, and then, the specimens were treated with anti-fluorescence quenching agent. The cells were observed with a fluorescence microscope (Olympus Japan). The number of tumor-infiltrating immune cells per high-power field (HPF, 10 × 40) of 3 replicates were counted independently HPF. Information on antibodies is in Supplementary Table S4. All results were assessed by 2 experienced pathologists.

### Statistical analyses

Statistical analyses were performed using SPSS 25.0 (IBM Corp., Armonk, NK, USA), GraphPad Prism 7 (GraphPad Software, Inc., La Jolla, CA, USA), and R software 4.2.1 (Lucent Technologies). For 2-sided tests, *P* < .05 was considered to indicate statistical significance. Results of assays with continuous variables are presented as medians and IQRs based on Student’s *t* or Mann-Whitney *U* tests. The relationship between *FGFR2* rearrangement and clinicopathological data was analyzed using 2-sided χ^2^ test results. Kaplan-Meier (K-M) and log-rank tests were performed for survival analyses. Univariate and multivariate Cox regression analyses were performed to determine the independent prognostic value on the basis of the clinicopathological data.

## Results

### Patient demographic and clinicopathological characteristics

**Figure 1.**
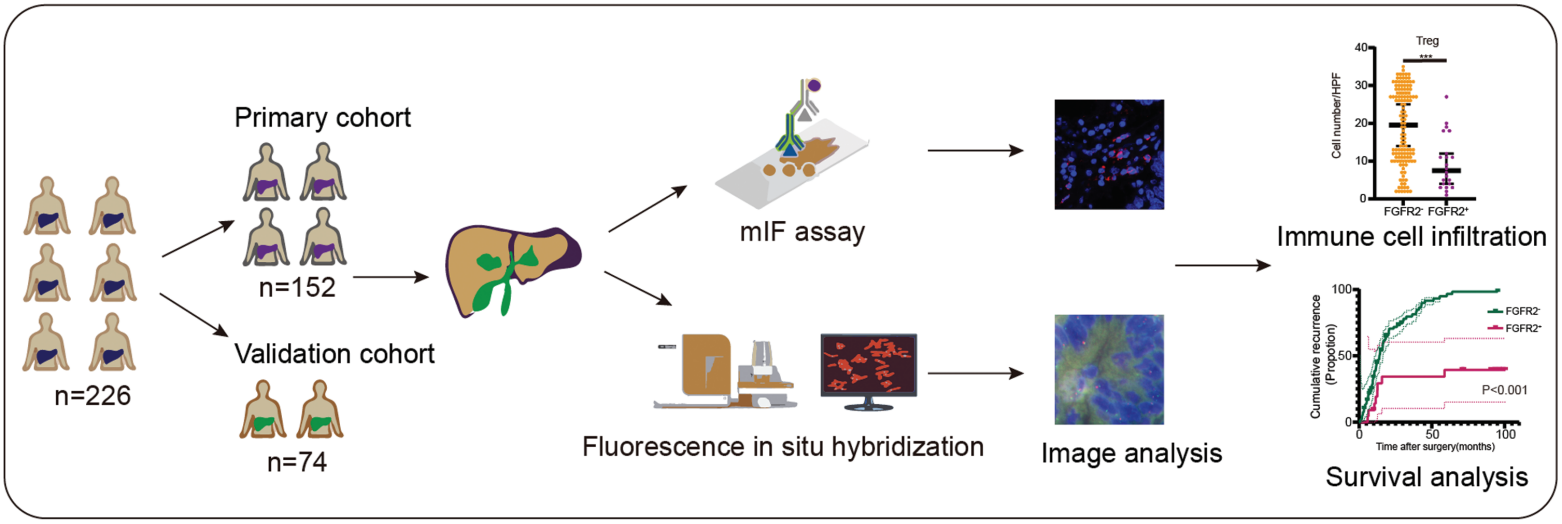
Overview of the prognosis and immune cells infiltration in ICC patients with and without FGFR2 fusion/rearrangement. ICC patients were assigned to 2 cohorts (primary cohort and validation cohort) groups, and samples were analyzed via FISH and multiplexed immunofluorescence (mIF) immunohistochemistry.

The flow chart showing the study steps is shown in Figure 1. In the primary cohort, the ages of the participants at initial diagnosis ranged from 31 to 85 years (median 59 years). A total of 62.5% patients (*n* = 95) were male, and 24.3% (*n* = 37) presented with clinical stage II disease, 59.8% (*n* = 91) exhibited abnormal CA-199 levels, and 25.0% (*n* = 38) suffered cirrhosis (Table 1). The validation cohort included 74 patients with ICC who met the criteria. The age distribution at initial diagnosis for participants in the validation ranged from 31 to 84 years (median 59 years). A total of 64.8% (*n* = 48) of these 74 patients were male, 22.9% (*n* = 17) presented with clinical stage II disease, 58.1% (*n* = 43) exhibited CA-199 levels that were higher than normal, and 35.1% (*n* = 26) showed signs of cirrhosis (Table 1). In addition, there were no significant differences between the primary and validation cohorts in any of the clinicopathologic characteristics except PD-L1 expression. (Table 1).

**Table 1.** The demographic and clinicopathological characteristics of patients with intrahepatic cholangiocarcinoma in the primary and validation cohort.

Variables	Primary cohort	Validation cohort	*P* value
Total	152	74	
Gender			
Male/female	95/57	48/26	.729
Age (years)			
≤55/>55	50/102	21/53	.492
CA-199			
≤34/>34 U/L	61/91	31/43	.800
CEA			
≤5/>5 ng/mL	108/44	52/22	.903
DBIL			
≤6.8/>6.8 ng/mL	129/23	59/15	.332
TBIL			
≤20.4/>20.4 ng/mL	139/13	66/8	.583
ALP			
≤125/>125 U/L	103/49	50/24	.976
AFP			
≤20/>20 ng/mL	142/10	64/10	.085
Albumin			
≤55/>55 g/L	74/78	36/38	.996
ALT			
≤50/>50 U/L	117/35	62/12	.237
γ-GGT			
≤60/>60 U/L	67/85	33/41	.942
Cirrhosis			
No/yes	114/38	48/26	.113
CNLC			
I/II/III	66/27/59	36/9/29	.526
TNM			
I/II/III/IV	66/55/28/3	36/25/11/2	.833
Differentiation			
I/II/III	14/61/77	4/27/43	.450
FGFR2 fusion/rearrangement			
No/yes	130/22	64/10	.846
PD-L1			
Negative/positive	97/55	31/43	**.002**
CSF1R			
Negative/positive	85/67	47/27	.277
Tumor-infiltrating Tregs, median (IQR)	16.0 (10.0-27.0)	14.5 (5.8-24.0)	.064
Tumor-infiltrating CD8^+^ T cells, median (IQR)	9.0 (3.3-23.6)	8.1 (3.1-31.6)	.602
Tumor-associated N1 neutrophils, median (IQR)	22.5 (10.1-43.4)	21.0 (8.9-39.1)	.390
Tumor-associated N2 neutrophils, median (IQR)	3.0 (1.0-6.0)	2.5 (1.0-5.6)	.739
Tumor-associated M1 macrophage, median (IQR)	4.5 (2.0-8.5)	5.0 (2.9-9.5)	.590
Tumor-associated M2 macrophage, median (IQR)	11.0 (4.1-21.0)	10.8 (5.0-24.3)	.746

Bold values represent statistical significance.

Abbreviations: CA-199, carbohydrate antigen 199; CEA, carcinoembryonic antigen; DBIL, direct bilirubin; TBIL, total bilirubin; ALP, alkaline phosphatase; AFP, alpha-fetoprotein; ALT, glutamic pyruvic transaminase; γ-GGT, gamma glutamyl transferase; CNLC, China liver cancer staging; FGFR2, fibroblast growth factor receptor 2; IQR, inter-quartile range.

### Relationship between *FGFR2* status and clinicopathological characteristics

**Figure 2.**
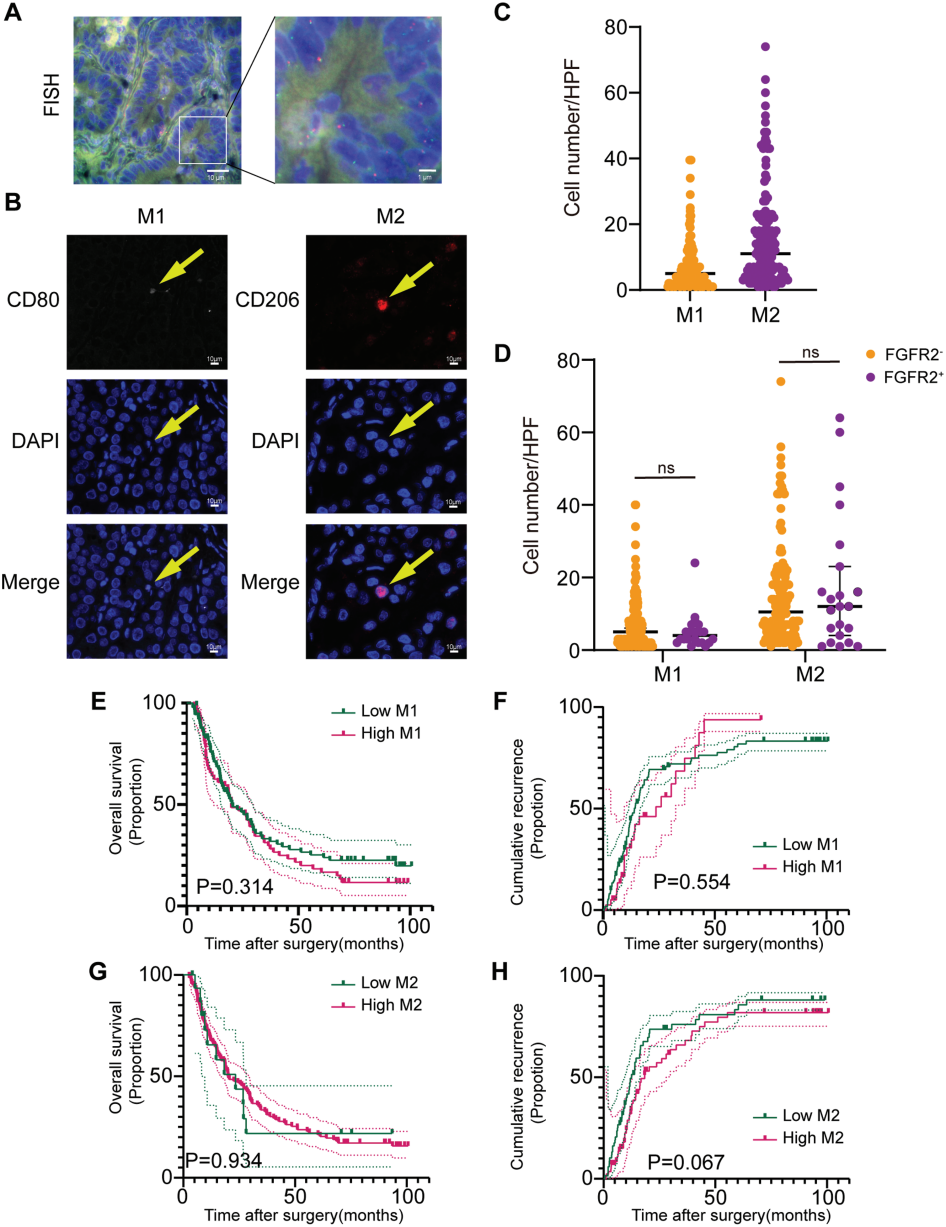
Correlation between *FGFR2* status and the infiltration of tumor-associated macrophages. (A) Representative images showing *FGFR2* fusion/rearrangement FISH staining in intrahepatic cholangiocarcinoma (ICC). (B) Representative images of mIF staining of tumor-associated M1/M2 macrophages. (C) Number of tumor-associated M1/M2 macrophage in the primary cohort. (D) Comparison of the number of tumor-associated M1/M2 macrophage in samples with different *FGFR2* statuses in the primary cohort samples. (E, F) Kaplan-Meier survival curve showing the OS and RFS according to the number of tumor-infiltrating M1 macrophages in the primary cohort. (G, H) Kaplan-Meier survival curve showing the OS and RFS according to the number of tumor-infiltrating M2 macrophages in the primary cohort.

The FISH results showed that 14.4% (*n* = 22) of the ICC patients in the primary cohort harbored *FGFR2* fusion/rearrangement (Figure 2A). As shown in Table 2, ICC patients with an *FGFR2* fusion/rearrangement presented with low levels of carcinoembryonic antigen (CEA, *P* = .026), gamma glutamyl transferase (γ-GGT, *P* = .003), and low CNCL/TNM staging. In addition, ICC patients with a concentration of CEA ≤ 5 ng/mL and γ-GGT ≤ 60 U/L tended to carry *FGFR2* fusion/rearrangement.

**Table 2.** Relationships between FGFR2 status and clinicopathological characteristics in the primary cohort.

Variables	FGFR2(−)	FGFR2(+)	*P* values
Gender			
Male/female	83/47	12/10	.405
Age (years)			
≤55/>55	42/88	8/14	.708
CA-199			
≤34/>34 U/L	49/81	12/10	.136
CEA			
≤5/>5 ng/mL	88/42	20/2	**.026**
DBIL			
≤6.8/>6.8 ng/mL	109/21	20/2	.594
TBIL			
≤20.4/>20.4 ng/mL	117/13	22/0	.255
ALP			
≤125/>125 U/L	85/45	18/4	.127
AFP			
≤20/>20 ng/mL	121/9	21/1	.677
Albumin			
≤55/>55 g/L	64/66	10/12	.743
ALT			
≤50/>50 U/L	98/32	19/3	.258
GGT			
≤60/>60 U/L	51/79	16/6	**.003**
Cirrhosis			
No/yes	97/33	17/5	.790
CNLC			
I/II/III	50/24/56	16/3/3	**.008**
TNM			
I/II/III/IV	50/49/28	16/16/0	**.012**
Differentiation			
I/II/III	10/52/68	4/9/9	.308
PD-L1			
Negative/positive	80/50	17/5	.158
CSF1R			
Negative/positive	75/55	10/12	.285
Tumor-infiltrating Tregs, median (IQR)	19.5 (11.0-28.3)	7.5 (3.8-13.5)	**<.001**
Tumor-infiltrating CD8^+^ T cells, median (IQR)	9.1 (3.5-27.1)	7.1 (2.4-22.4)	.821
Tumor-associated N1 neutrophils, median (IQR)	18.5 (7.0-33.5)	53.5 (44.4-56.8)	**<.001**
Tumor-associated N2 neutrophils, median (IQR)	3.0 (1.0-6.5)	1.3 (0.5-4.0)	**<.001**
Tumor-associated M1 macrophage, median (IQR)	4.8 (2.0-9.3)	3.8 (2.0-5.4)	.063
Tumor-associated M2 macrophage, median (IQR)	10.3 (4.4-21.0)	11.8 (3.1-24.0)	.473

Bold values represent statistical significance.

Abbreviations: CA-199, carbohydrate antigen 199; CEA, carcinoembryonic antigen; DBIL, direct bilirubin; TBIL, total bilirubin; ALP, alkaline phosphatase; AFP, alpha-fetoprotein; ALT, glutamic pyruvic transaminase; γ-GGT, gamma glutamyl transferase; CNLC, China liver cancer staging; FGFR2, fibroblast growth factor receptor 2; IQR, inter-quartile range.

### *FGFR2* fusion/rearrangement fosters an immunoactivated microenvironment in ICC tissues

To further investigate the relationship between *FGFR2* status and the tumor immune microenvironment, we profiled the immune cells that infiltrated into ICC tissues using mIF and IHC assays. In the primary cohort, the median numbers of M1 macrophages and M2 macrophages were 4.5 (IQR 2.0-8.5) and 11.0 (IQR 4.1-21.0), respectively (Table 1; Figure 2B, 2C). A correlation analysis showed that *FGFR2* status did not correlate with tumor-associated macrophages (TAMs; *P* > .05; Table 2; Figure 2D). A K-M analysis revealed no significant difference in median OS and RFS between the patients in the high- and low-M1 macrophage infiltration and between the patients in the high- and low-M2 macrophage infiltration groups (M1 macrophage-infiltrating groups, OS: *P* = .314, Figure 2E; RFS: *P* = .554, Figure 2F) and M2 macrophage-infiltrating groups (OS: *P* = .934, Figure 2G; RFS: *P* = .067, Figure 2H).

**Figure 3.**
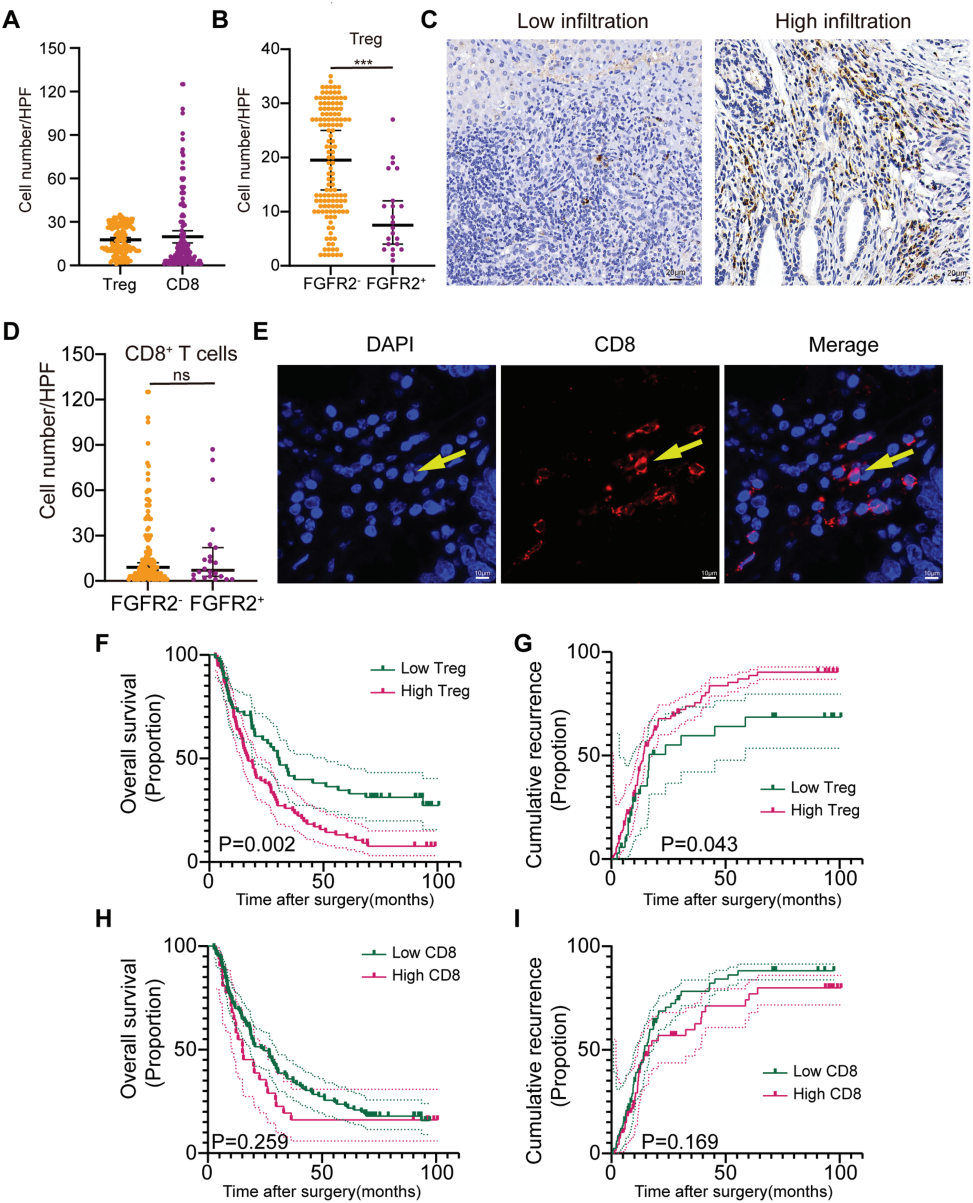
Correlation between *FGFR2* status and the infiltration of Tregs or CD8^+^ T cells. (A) The number of tumor-infiltrating Tregs or CD8^+^ T cells in the primary cohort. (B) Comparison of the number of tumor-infiltrating Tregs in the primary cohort samples with different *FGFR2* statuses. (C) Representative images showing the immunohistochemistry of tumor-infiltrating Tregs. (D) Comparison of the number of tumor-infiltrating CD8^+^ T cells in tissues with different *FGFR2* statuses in the primary cohort. (E) Representative images showing mIF staining for tumor-infiltrating CD8^+^ T cells. (F, G) Kaplan-Meier survival curve showing the OS and RFS according to the number of tumor-infiltrating Tregs in the primary cohort. (H, I) Kaplan-Meier survival curve showing the OS and RFS according to number of tumor-infiltrating CD8+ T cells in the primary cohort.

The median numbers of tumor-infiltrating CD8^+^ T cells and Tregs in the primary cohort were 9.0 (IQR 3.3-23.6) and 16.0 (IQR 10.0-27.0), respectively (Table 1; Figure 3A). A correlation analysis showed that the number of tumor-infiltrating Treg cells was lower in the ICC patients with an *FGFR2* fusion/rearrangement (*P* < .001; Table 2; Figure 3B, 3C), but there was no difference in the number of infiltrating CD8^+^ T cells (*P* = .821; Table 2; Figure 3D, 3E). A K-M analysis showed that ICC patients for whom Tregs were highly infiltrated into cancer tissue (OS: *P* = .002, Figure 3F; RFS: *P* = .043, Figure 3G) were associated with poor OS and RFS; however, CD8^+^ T-cell infiltration was not correlated with prognosis (OS: *P* = .259, Figure 3H; RFS: *P* = .169, Figure 3I).

**Figure 4.**
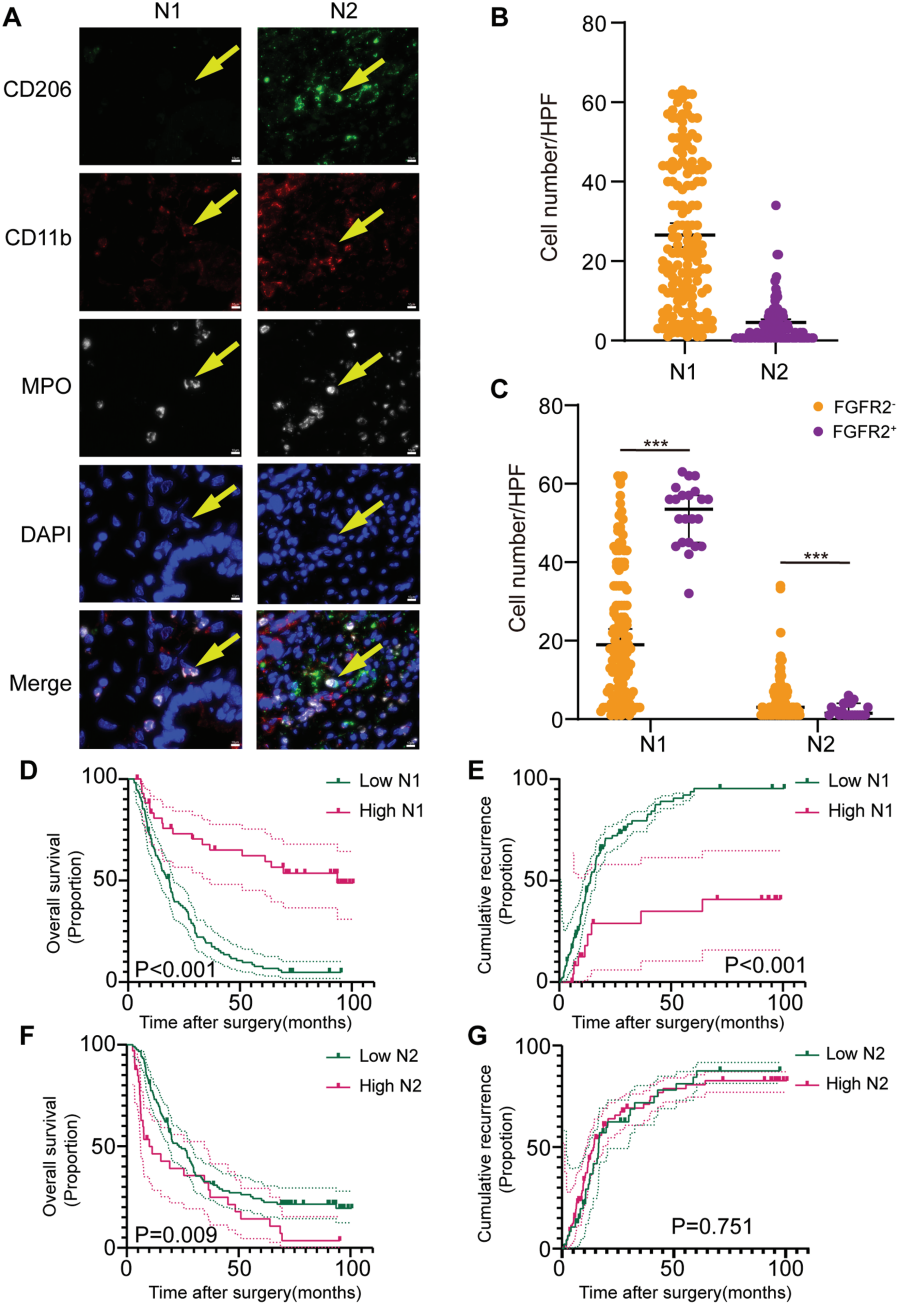
Correlation between *FGFR2* status and infiltration of tumor-associated neutrophils. (A) Representative images showing mIF staining of tumor-associated N1/N2 neutrophils. (B) Number of tumor-associated neutrophil in the primary cohort. (C) Comparison of the number of tumor-associated neutrophils in the primary cohort samples with different *FGFR2* statuses. (D, E) Kaplan-Meier survival curve showing the OS and RFS according to the number of tumor-infiltrating N1 neutrophils in the primary cohort. (F, G) Kaplan-Meier survival curve showing the OS and RFS according to the number of tumor-infiltrating N2 neutrophils in the primary cohort.

The median numbers of tumor-infiltrating N1 neutrophils and N2 neutrophils in the primary cohort were 22.5 (IQR 10.1-43.4) and 3.0 (IQR 1.0-6.0), respectively (Table 1; Figure 4A, 4B). The results of a correlation analysis suggested that the number of tumor-infiltrating N1 neutrophils was high (*P* < .001) and that the number of N2 neutrophils was low (*P* < .001) in ICC patients with an *FGFR2* fusion/rearrangement (Table 2; Figure 4C). A K-M analysis suggested that ICC patients with a high level of infiltrated N1 neutrophils (OS: *P* < .001, Figure 4D; RFS: *P* < .001, Figure 4E) were associated with favorable OS and RFS, whereas N2 neutrophil infiltration was associated with poor OS but not with RFS (OS: *P* = .009, Figure 4F; RFS: *P* = .751, Figure 4G).

PD-L1 and CSF1R are 2 important immune-related proteins. They were divided into high and low expression groups based on IHC results (Supplementary Figure S1A, S1B). The results of correlation analysis suggested that FGFR2 status did not correlate with PD-L1 and CSF1R expression (Table 2; Supplementary Figure S1C). K-M analysis suggested that CSF1R expression was not correlated with the prognosis of ICC patients (Supplementary Figure S1D, S1E); whereas high expression of PD-L1 was associated with poor OS (P = .01), but not RFS (Supplementary Figure S1F, S1G). These results reveal the immune profile of ICC tissues with different *FGFR2* statuses.

### *FGFR2* fusion/rearrangement was correlated with favorable prognosis in ICC patients

**Figure 5.**
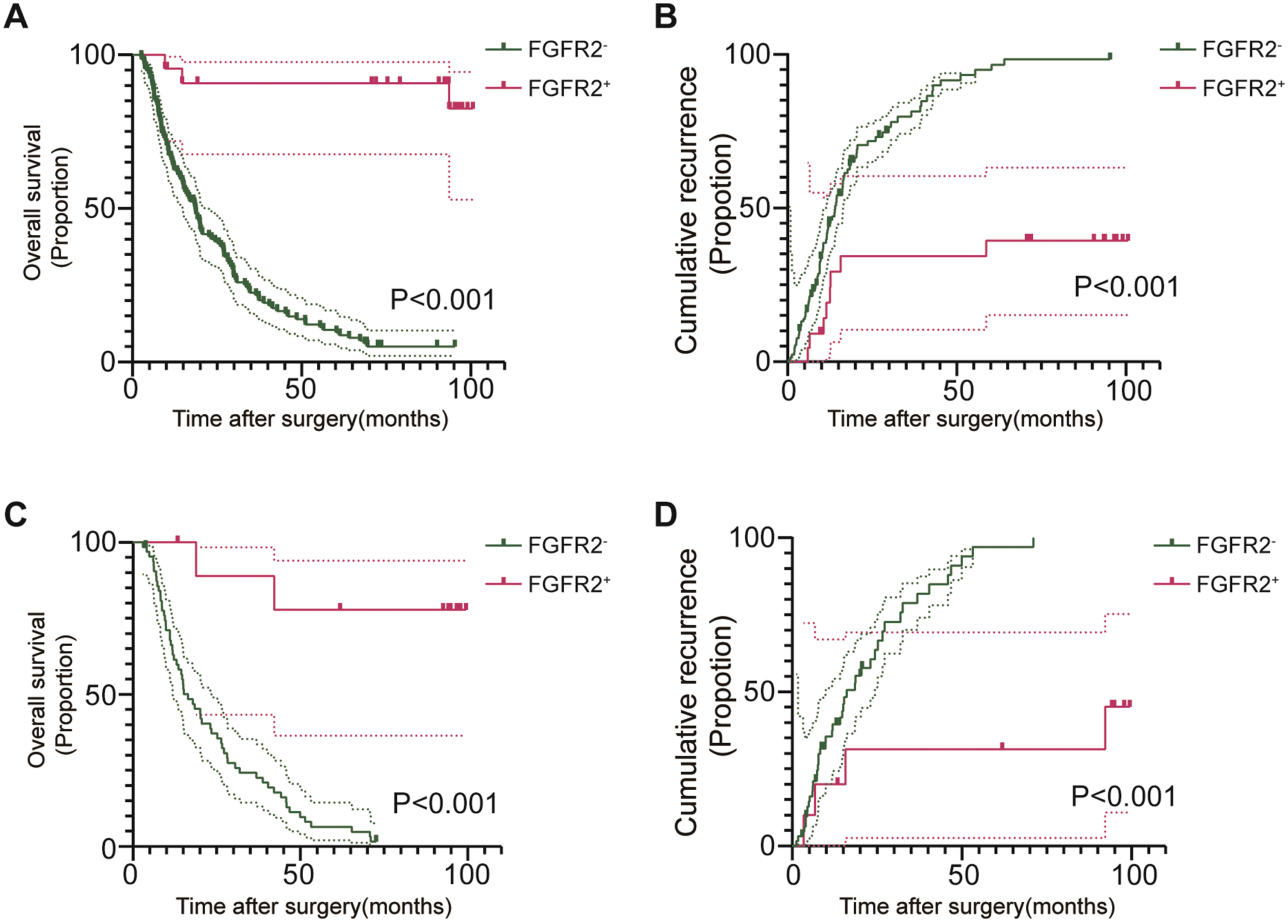
*FGFR2* fusion/rearrangement mutation leads to a poor prognosis in patients with ICC. (A, B) Kaplan-Meier survival curve showing the OS and RFS according to the *FGFR2* status in the primary cohort samples. (C, D) Kaplan-Meier survival curve showing the OS and RFS according to *FGFR2* status in the validation cohort samples.

In the primary cohort, at a median follow-up of 11.1 months (0.56-100.6 months), 63.8% (*n* = 97) of the patients had suffered from relapse. Additionally, at a median follow-up of 19.2 months (2.6-100.6 months), 78.9% (*n* = 120) of patients had died. A K-M analysis was performed and multivariate COX regression models were established to further clarify the prognostic relevance of *FGFR2* fusion/rearrangement. The K-M analysis suggested that ICC patients with an *FGFR2* fusion/rearrangement were associated with a favorable OS and RFS (Figure 5A, 5B). *FGFR2* fusion/rearrangement reduced the risk of death 0.89-fold (*P* = .002; Table 3). Moreover, high levels of CEA (*P* < .001), TBIL (*P* = .026), PD-L1 expression (P < .001), and CNLC (*P* = .024), and low levels of albumin (*P* = .036) and tumor-infiltrating N1 neutrophils (*P* = .019) were associated with a shortened OS time (Table 3). High levels of AFP (*P* = .002) and low levels of tumor-infiltrating N1 neutrophils (*P* = .006) were associated with poor RFS (Table 4). Collectively, these data enabled us to determine the value of *FGFR2* fusion/rearrangement in predicting the prognosis of ICC.

**Table 3.** Univariate and multivariate analysis of clinicopathological characteristics with overall survival in the primary cohort.

Variables	Multivariate HR (95% CI)	Univariate *P* value	Multivariate HR (95% CI)	Multivariate *P* value
Gender				
Male/female	1.214 (0.838-1.758)	.306	NA	
Age (years)				
≤55/>55	1.286 (0.881-1.877)	.193	NA	
CA-199				
≤34/>34U/L	1.484 (1.027-2.145)	**.036**	0.770 (0.497-1.194)	.242
CEA				
≤5/>5 ng/mL	2.356 (1.592-3.486)	**<.001**	2.946 (1.814-4.786)	**<.001**
DBIL				
≤6.8/>6.8 ng/mL	1.535 (0.958-2.459)	.075	NA	
TBIL				
≤20.4/>20.4 ng/mL	2.192 (1.230-3.907)	**.008**	2.375 (1.110-5.080)	**.026**
ALP				
≤125/>125 U/L	1.576 (1.086-2.287)	**.017**	1.181 (0.712-1.959)	.520
AFP				
≤20/>20 ng/mL	1.888 (0.985-3.619)	.056	NA	
Albumin				
≤55/>55g/L	0.675 (0.471-0.967)	**.032**	0.650 (0.435-0.972)	**.036**
ALT				
≤50/>50U/L	1.177 (0.778-1.781)	.440	NA	
GGT				
≤60/>60U/L	1.611 (1.112-2.333)	**.012**	1.111 (0.700-1.761)	.656
Cirrhosis				
No/yes	0.823 (0.539-1.257)	.368	NA	
CNLC				
I/II/III	1.850 (1.509-2.267)	**<.001**	1.677 (1.071-2.626)	**.024**
TNM				
I/II/III/IV	2.072 (1.668-2.574)	**<.001**	1.388 (0.868-2.218)	.171
Differentiation				
I/II/III	1.365 (1.029-1.811)	**.031**	1.283 (0.933-1.765)	.125
FGFR2 fusion				
No/yes	0.051 (0.016-0.165)	**<.001**	0.113 (0.029-0.445)	**.002**
PD-L1				
Negative/positive	1.617 (1.119-2.337)	**.010**	2.572 (1.703-3.884)	**<.001**
CSF1R				
Negative/positive	0.838 (0.583-1.204)	.340	NA	
Tumor-infiltrating Tregs				
Low/high	1.836 (1.254-2.687)	**.002**	1.196 (0.803-1.780)	.379
Tumor-infiltrating CD8^+^ T cells				
Low/high	1.284 (0.830-1.985)	.261	NA	
Tumor-associated N1 neutrophils				
Low/high	0.237 (0.142-0.396)	**<.001**	0.491 (0.271-0.890)	**.019**
Tumor-associated N2 neutrophils				
Low/high	1.738 (1.143-2.644)	**.010**	1.236 (0.743-2.054)	.414
Tumor-associated M1 macrophage				
Low/high	1.202 (0.839-1.722)	.315	NA	
Tumor-associated M2 macrophage				
Low/high	0.974 (0.523-1.813)	.934	NA	

Bold values represent statistical significance.

Abbreviations: CA-199, carbohydrate antigen 199; CEA, carcinoembryonic antigen; DBIL, direct bilirubin; TBIL, total bilirubin; ALP, alkaline phosphatase; AFP, alpha-fetoprotein; ALT, glutamic pyruvic transaminase; γ-GGT, gamma glutamyl transferase; CNLC, China liver cancer staging; FGFR2, fibroblast growth factor receptor 2; IQR, inter-quartile range.

**Table 4.** Univariate and multivariate analysis of clinicopathological characteristics with recurrence-free survival in the primary cohort.

Variables	Multivariate HR (95% CI)	Univariate *P* value	Multivariate HR (95% CI)	Multivariate *P* value
Gender				
Male/female	1.556 (1.021-2.372)	**.040**	1.450 (0.934-2.251)	.097
Age (years)				
≤55/>55	1.023 (0.675-1.550)	.914	NA	
CA-199				
≤34/>34U/L	1.406 (0.934-2.119)	.104	NA	
CEA				
≤5/>5 ng/mL	1.626 (1.025-2.581)	**.039**	1.274 (0.780-2.080)	.334
DBIL				
≤6.8/>6.8 ng/mL	1.466 (0.839-2.559)	.179	NA	
TBIL				
≤20.4/>20.4 ng/mL	1.337 (0.647-2.762)	.433	NA	
ALP				
≤125/>125 U/L	1.791 (1.185-2.708)	**.006**	1.436 (0.899-2.294)	.130
AFP				
≤20/>20 ng/mL	3.233 (1.449-7.214)	**.004**	3.929 (1.645-9.380)	**.002**
Albumin				
≤55/>55g/L	1.226 (0.817-1.838)	.325	NA	
ALT				
≤50/>50 U/L	1.388 (0.885-2.179)	.154	NA	
GGT				
≤60/>60 U/L	1.748 (1.156-2.643)	**.008**	1.240 (0.767-2.007)	.380
Cirrhosis				
No/yes	1.334 (0.854-2.084)	.205	NA	
CNLC				
I/II/III	1.285 (1.024-1.613)	**.031**	0.882 (0.524-1.486)	.638
TNM				
I/II/III/IV	1.286 (1.004-1.647)	**.047**	1.324 (0.733-2.391)	.352
Differentiation				
I/II/III	1.318 (0.961-1.808)	.087	NA	
FGFR2 fusion/rearrangement				
No/yes	0.179 (0.083-0.386)	**<.001**	0.523 (0.196-1.396)	.196
PD-L1				
Negative/positive	1.335 (0.877-2.035)	.178	NA	
CSF1R				
Negative/positive	0.923 (0.619-1.378)	.696	NA	
Tumor-infiltrating Tregs				
Low/high	1.675 (1.010-2.778)	**.046**	1.275 (0.712-2.282)	.413
Tumor-infiltrating CD8^+^ T cells				
Low/high	0.752 (0.501-1.130)	.171	NA	
Tumor-associated N1 neutrophils				
Low/high	0.210 (0.099-0.444)	**<.001**	0.302 (0.128-0.709)	**.006**
Tumor-associated N2 neutrophils				
Low/high	1.070 (0.704-1.626)	.751	NA	
Tumor-associated M1 macrophage				
Low/high	0.866 (0.536-1.397)	.554	NA	
Tumor-associated M2 macrophage				
Low/high	0.691 (0.464-1.029)	.069	NA	

Bold values represent statistical significance.

Abbreviations: CA-199, carbohydrate antigen 199; CEA, carcinoembryonic antigen; DBIL, direct bilirubin; TBIL, total bilirubin; ALP, alkaline phosphatase; AFP, alpha-fetoprotein; ALT, glutamic pyruvic transaminase; γ-GGT, gamma glutamyl transferase; CNLC, China liver cancer staging; FGFR2, fibroblast growth factor receptor 2; IQR, inter-quartile range.

### Validation of *FGFR2* status along with immune infiltration and survival

In the validation cohort, 13.5% (*n* = 10) of the ICC patients presented with an *FGFR2* fusion/rearrangement. As shown in Supplementary Table S1, *FGFR2* status was significantly correlated with cell differentiation (*P* = .016). The median number of tumor-infiltrating Tregs and CD8^+^ T cells was 14.5 (IQR 5.8-24.0) and 8.1 (IQR 3.1-31.6), respectively; the median number of tumor-associated N1 and N2 neutrophils was 21.0 (IQR 8.9-39.1) and 2.5 (IQR 1.0-5.6), respectively; and the median number of tumor-associated M1 and M2 macrophages was 5.0 (IQR 2.9-9.5) and 10.8 (IQR 5.0-24.3), respectively (Table 1; Supplementary Figure S2). The results of a correlation analysis suggested that *FGFR2* fusion/rearrangement was associated with low levels infiltrating Tregs (*P* = .031), low levels of infiltrating N2 neutrophils (*P* = .002) and high levels of infiltrating N1 neutrophils (*P* < .001; Supplementary Table S1; Supplementary Figure S2).

At a median follow-up of 13.2 months (1.2-99.4 months), 66.2% (*n* = 49) of the patients had experienced relapse. Additionally, at a median follow-up of 18.6 months (2.9-99.4 months), 85.1% (*n* = 63) of the patients had died. The prognostic analysis suggested that an *FGFR2* fusion/rearrangement reduced the risk of death by 0.96-fold (*P* = .002) and the risk of recurrence by 0.85-fold (*P* = .006; Supplementary Tables S2 and S3; Figure 5C, 5D). Additionally, high levels of ALP (*P* = .018) and PD-L1 (*P* = .038) were associated with poor OS (Supplementary Table S2). A K-M prognostic analysis suggested that high levels of tumor-infiltrating Tregs (*P* = .032), low levels of tumor-infiltrating N1 neutrophils (*P* < .001), high levels of tumor-infiltrating M2 macrophages (*P* = .016) and high PD-L1 expression (*P* = .002) were associated with poor OS, while a high level of infiltrating Tregs was associated with shortened RFS time (*P* = .011; Supplementary Figures S1 and S3). Taken together, these findings emphasize the independence and reliability of *FGFR2* fusion/rearrangement in predicting prognosis and its association with the immune context.

## Discussion

Our study revealed a correlation between clinicopathological features and immune cell infiltration into tumors with an *FGFR2* fusion/rearrangement. Then, we assessed the prognostic value of the variants. According to our results, *FGFR2* fusion/rearrangement is an independent prognostic factor and is closely associated with immune cell infiltration in ICC tumors. This work helps us better understand the role of *FGFR2* fusion/rearrangement in patients with ICC and provides us with a new perspective on ways to personalize the treatment of ICC patients with an *FGFR2* fusion/rearrangement.

With recent advances in next-generation sequencing technology, an increasing number of mutated genes, such as Kirsten rat sarcoma viral oncogene homolog (KRAS), isocitrate dehydrogenase (IDH) and *FGFR2* mutations, have been identified as prognostic and therapeutic targets for people with ICC.^10,16^*FGFR2* fusion/rearrangement had previously been reported to be found in 10%-16% of all ICC patients; similarly, 14.2% of the patients in this study harbored an *FGFR2* fusion/rearrangement.^10,14,17^ Next-generation sequencing technology revealed multiple *FGFR2* fusion partners, including BICC1, PHLN1, AHCYL1, and TACC3.^16^ Previous studies revealed a correlation between patient age and HCV/HBV infection or *FGFR2* rearrangement and a higher incidence of *FGFR2* rearrangement in ICC patients 65 years of age or younger or who were HCV/HBV positive.^18^ However, these correlations were found in the present study. Churi et al^19^ found that FGFR genetic aberrations were associated with better prognosis in cholangiocarcinoma; they examined the mutation profile of cholangiocarcinoma and found that the median OS of *FGFR2* rearrangement-positive patients was 38.8 months. Our study revealed that *FGFR2* fusion/rearrangement were associated with prolonged OS and RFS times. These findings suggest that the clinical characteristics of *FGFR2* fusion/rearrangement are associated with clinical benefit, but further validation with a large group of clinical samples is needed.

ICC is now the second most common type of primary liver cancer after HCC, and its increasing incidence and poor prognosis have made it a major health concern.^20^ Serum albumin level is one of the most important indicators of the nutritional status of the body. Nutrition-related marker levels are currently measured as important prognostic indicators after ICC resection.^21^ The present study also showed that high preoperative serum albumin levels were associated with good OS in ICC patients but not with RFS. CEA is another important tumor marker in the digestive system. There is some controversy about the relationship between CEA and ICC prognosis in previous studies. In this study, high serum CEA levels were found to be associated with poor OS but not RFS. In any case, a larger sample is still needed to validate these results.

The TME plays an important role in ICC growth and metastasis, with immune cells exerting particularly important effects.^22^ Previous studies have shown that FoxP3^+^ Treg infiltration was associated with poor RFS in patients with ICC and was a favorable factor in the pathogenesis of ICC patients.^23^ More immunosuppressive CD4+ Tregs were infiltrated in ICC compared to peritumor, where MEOX1 was highly enriched in tumor-infiltrating Tregs.^24^ In this study, we found that FoxP3^+^ Treg infiltration was low in the *FGFR2* fusion/rearrangement ICC group, suggesting that the association of *FGFR2* fusion/rearrangement with good prognosis in ICC may be due to a reduced Treg infiltration rate. CD8 is a cytotoxic T-cell surface marker. Intratumor immune status influences tumor response to therapy, and high CD8 GZMB^+^ and CD8 proliferation ratios suggest a good therapeutic response to ICC.^25^ Many studies have implied that high levels of CD8^+^ T cell infiltrating into tumor tissues is associated with a positive prognosis, but the opposite result has been observed in ICC patients.^26^ In this study, no prognostic correlation between CD8^+^ T-cell infiltration and ICC was observed, and *FGFR2* mutations were not associated with CD8^+^ T-cell infiltration. These results suggest that *FGFR2* mutations may inhibit the activation of CD8^+^ T cells. Macrophages play important roles in innate immunity and can be classified as M1 macrophages and M2 macrophages according to their function. Consistent with the results of previous studies, the present study revealed a preference for M2 macrophage compared with M1 macrophage infiltration in ICC tissues.^23^ However, no prognostic correlation between macrophage infiltration and ICC was observed in this study, and *FGFR2* mutations were not associated with macrophage infiltration. These outcomes suggest that *FGFR2* mutations may exert little effect on macrophage polarization. Neutrophils, which are involved in of innate immunity, can promote tumor cell proliferation and metastasis.^27^ Neutrophils can be classified into N1 neutrophils and N2 neutrophils according to their surface markers.^28^ A previous study showed that high levels of infiltrated neutrophils were associated with poor prognosis in pancreatic ductal adenocarcinoma.^28^Neutrophil extracellular traps promote the motility and migration of ICC cells in vitro.^29^ Furthermore, TAM and tumor-associated neutrophils interact to promote ICC progression through activation of the STAT3 pathway.^30^ In our study, N1 neutrophils are associated with a favorable prognosis for patients with ICC. Meanwhile, the results of correlation between *FGFR2* fusion/rearrangement and neutrophil infiltration indicated that N1 neutrophil infiltration was high and that N2 neutrophil infiltration was low in *FGFR2* fused/rearranged ICC tissues. These results suggest that *FGFR2* mutations may be involved in inducing neutrophil polarization and may affect prognosis, but further experiments are needed to verify these possibilities.

There are some limitations in this study. First, this was a single-center retrospective clinical study, and therefore, the data may be biased. Moreover, we did not elucidate the specific molecular mechanisms underlying the differences in immune cell infiltration due to *FGFR2* mutations. Therefore, multicenter, large-scale, prospective, and molecular studies are still needed to explore the relationship between *FGFR2* and ICC prognosis and immune infiltration in the future.

## Conclusion

*FGFR2* fusion/rearrangement are associated with good clinical outcomes and an immune-activated tumor microenvironment in patients with ICC. *FGFR2* status may be used for ICC prognostic stratification of patients and as a potential immunotherapeutic target for ICC.

## Supplementary material

Supplementary material is available at *The Oncologist* online.

oyae170_suppl_Supplementary_Tables

oyae170_suppl_Supplementary_Figures

## Data Availability

The data underlying this article will be shared on reasonable request to the corresponding author.
