# Supplementary material for: FGFR2 fusion/rearrangement is associated with favorable prognosis and immunoactivation in patients with intrahepatic cholangiocarcinoma
Source: Oncologist. 2024 Jul 10;29(12):e1734–47. doi: 10.1093/oncolo/oyae170 (PMC11630758; doi:10.1093/oncolo/oyae170)
Supplement: oyae170_suppl_Supplementary_Tables [file oyae170_suppl_supplementary_tables.docx]

***FGFR2* fusion/rearrangement is associated with favorable prognosis and immunoactivation in patients with intrahepatic cholangiocarcinoma**

**Supplementary Table 1** Relationships between FGFR2 status and clinicopathological characteristics in the validation cohort

| **Variables** | **FGFR2(-)** | **FGFR2(+)** | **P values** |
| --- | --- | --- | --- |
| Gender |  |  | 1.000 |
| Male/female | 41/23 | 7/3 |  |
| Age(years) |  |  | 1.000 |
| ≤55/>55 | 18/46 | 3/7 |  |
| CA-199 |  |  | 0.083 |
| ≤34/>34U/L | 24/40 | 7/3 |  |
| CEA |  |  | 0.264 |
| ≤5/>5ng/mL | 43/21 | 9/1 |  |
| DBIL |  |  | 0.110 |
| ≤6.8/>6.8ng/mL | 53/11 | 6/4 |  |
| TBIL |  |  | 1.000 |
| ≤20.4/>20.4ng/mL | 57/7 | 9/1 |  |
| ALP |  |  | 0.152 |
| ≤125/>125 | 41/23 | 9/1 |  |
| AFP |  |  | 0.339 |
| ≤20/>20 | 54/10 | 10/0 |  |
| Albumin |  |  | 0.737 |
| ≤55/>55g/L | 32/32 | 4/6 |  |
| ALT |  |  | 0.200 |
| ≤50/>50U/L | 52/12 | 10/0 |  |
| GGT |  |  | 0.326 |
| ≤60/>60U/L | 27/37 | 6/4 |  |
| Cirrhosis |  |  | 0.734 |
| No/yes | 42/22 | 6/4 |  |
| CNLC |  |  | 0.131 |
| Ia/Ib/IIa/IIb | 23/26/6/9 | 6/2/2/0 |  |
| Differentiation |  |  | **0.016** |
| I/II/III | 2/21/41 | 2/6/2 |  |
| Tumor-infiltrating Tregs, median(IQR) | 15.5(8.0-25.0) | 6.0(3.8-13.8) | **0.031** |
| Tumor-infiltrating CD8^+^ T cells, median(IQR) | 8.8(3.3-32.9) | 6.25(2.5-29.8) | 0.485 |
| Tumor-associated N1 neutrophils, median (IQR) | 18.0(6.0-32.4) | 51.5(50.1-56.0) | **<0.001** |
| Tumor-associated N2 neutrophils, median (IQR) | 2.5(1.0-6.0) | 2.3(0.5-3.1) | **0.002** |
| Tumor-associated M1 macrophage, median (IQR) | 5.0(2.6-9.5) | 5.00(2.9-8.9) | 0.679 |
| Tumor-associated M2 macrophage, median (IQR) | 11.5(5.1-25.4) | 9.5(3.4-22.1) | 0.354 |

Abbreviations: CA-199, carbohydrate antigen 199; CEA, carcinoembryonic antigen; DBIL, direct bilirubin; TBIL, total bilirubin; ALP, alkaline phosphatase; AFP, alpha-fetoprotein; ALT, glutamic pyruvic transaminase; γ-GGT, gamma glutamyl transferase; CNLC, China liver cancer staging; FGFR2, fibroblast growth factor receptor 2; IQR, inter-quartile range.

Supplementary Table 2 Univariate and multivariate analysis of clinicopathological characteristics with overall survival in the validation cohort

| **Variables** | **Multivariate**  **HR(95%CI)** | **Univariate**  **P value** | **Multivariate**  **HR(95%CI)** | **Multivariate**  **P value** |
| --- | --- | --- | --- | --- |
| Gender |  |  |  |  |
| Male/female | 0.787(0.472-1.313) | 0.359 | NA |  |
| Age(years) |  |  |  |  |
| ≤55/>55 | 0.936(0.541-1.619) | 0.813 | NA |  |
| CA-199 |  |  |  |  |
| ≤34/>34U/L | 2.142(1.269-3.617) | **0.004** | 1.747(0.981-3.112) | 0.058 |
| CEA |  |  |  |  |
| ≤5/>5ng/mL | 2.041(1.201-3.468) | **0.008** | 0.955(0.502-1.817) | 0.888 |
| DBIL |  |  |  |  |
| ≤6.8/>6.8ng/mL | 0.684(0.356-1.314) | 0.254 | NA |  |
| TBIL |  |  |  |  |
| ≤20.4/>20.4ng/mL | 2.054(0.921-4.582) | 0.079 | NA |  |
| ALP |  |  |  |  |
| ≤125/>125 | 2.949(1.707-5.094) | **<0.001** | 2.213(1.149-4.266) | **0.018** |
| AFP |  |  |  |  |
| ≤20/>20 | 1.384(0.699-2.740) | 0.351 | NA |  |
| Albumin |  |  |  |  |
| ≤55/>55g/L | 0.700(0.426-1.151) | 0.160 | NA |  |
| ALT |  |  |  |  |
| ≤50/>50U/L | 1.674(0.860-3.259) | 0.130 | NA |  |
| GGT |  |  |  |  |
| ≤60/>60U/L | 1.583(0.958-2.616) | 0.073 | NA |  |
| Cirrhosis |  |  |  |  |
| No/yes | 0.858(0.513-1.437) | 0.561 | NA |  |
| CNLC |  |  |  |  |
| I/II/III | 1.302(0.997-1.699) | 0.052 | NA |  |
| TNM |  |  |  |  |
| I/II/III/IV | 1.248(0.939-1.658) | 0.127 | NA |  |
| Differentiation |  |  |  |  |
| I/II/III | 1.626(1.037-2.549) | **0.034** | 1.369(0.776-2.415) | 0.278 |
| FGFR2 fusion |  |  |  |  |
| No/yes | 0.074(0.018-0.308) | **<0.001** | 0.045(0.007-0.310) | **0.002** |
| PD-L1 |  |  |  |  |
| Negative/Positive | 2.263(1.338-3.825) | **0.002** | 1.957(1.037-3.691) | **0.038** |
| CSF1R |  |  |  |  |
| Negative/Positive | 1.260(0.761-2.088) | 0.369 | NA |  |
| Tumor-infiltrating Tregs |  |  |  |  |
| Low/high | 2.243(1.052-4.775) | **0.036** | 0.704(0.309-1.605) | 0.404 |

Continued

Supplementary Table 2 Continued

| **Variables** | **Multivariate**  **HR(95%CI)** | **Univariate**  **P value** | **Multivariate**  **HR(95%CI)** | **Multivariate**  **P value** |
| --- | --- | --- | --- | --- |
| Tumor-infiltrating CD8^+^ T cells |  |  |  |  |
| Low/high | 0.877(0.445-1.728) | 0.704 | NA |  |
| Tumor-associated N1 neutrophils |  |  |  |  |
| Low/high | 0.198(0.079-0.500) | **0.001** | 1.919(0.540-6.816) | 0.314 |
| Tumor-associated N2 neutrophils |  |  |  |  |
| Low/high | 1.465(0.865-2.482) | 0.156 | NA |  |
| Tumor-associated M1 macrophage |  |  |  |  |
| Low/high | 0.830(0.458-1.506) | 0.541 | NA |  |
| Tumor-associated M2 macrophage |  |  |  |  |
| Low/high | 2.074(1.143-3.763) | **0.016** | 1.245(0.667-2.323) | 0.491 |

Abbreviations: CA-199, carbohydrate antigen 199; CEA, carcinoembryonic antigen; DBIL, direct bilirubin; TBIL, total bilirubin; ALP, alkaline phosphatase; AFP, alpha-fetoprotein; ALT, glutamic pyruvic transaminase; γ-GGT, gamma glutamyl transferase; CNLC, China liver cancer staging; FGFR2, fibroblast growth factor receptor 2; IQR, inter-quartile range.

Supplementary Table 3 Univariate and multivariate analysis of clinicopathological characteristics with recurrence-free survival in the validation cohort

| **Variables** | **Multivariate**  **HR(95%CI)** | **Univariate**  **P value** | **Multivariate**  **HR(95%CI)** | **Multivariate**  **P value** |
| --- | --- | --- | --- | --- |
| Gender |  |  |  |  |
| Male/female | 1.042(0.576-1.886) | 0.891 | NA |  |
| Age(years) |  |  |  |  |
| ≤55/>55 | 0.808(0.433-1.506) | 0.502 | NA |  |
| CA-199 |  |  |  |  |
| ≤34/>34U/L | 1.687(0.944-3.014) | 0.077 | NA |  |
| CEA |  |  |  |  |
| ≤5/>5ng/mL | 1.790(0.979-3.271) | 0.058 | NA |  |
| DBIL |  |  |  |  |
| ≤6.8/>6.8ng/mL | 0.821(0.414-1.630) | 0.573 | NA |  |
| TBIL |  |  |  |  |
| ≤20.4/>20.4ng/mL | 2.618(1.081-6.338) | **0.033** | 1.279(0.459-3.562) | 0.638 |
| ALP |  |  |  |  |
| ≤125/>125 | 1.904(1.016-3.568) | **0.044** | 1.372(0.642-2.934) | 0.414 |
| AFP |  |  |  |  |
| ≤20/>20 | 1.206(0.506-2.875) | 0.672 | NA |  |
| Albumin |  |  |  |  |
| ≤55/>55g/L | 1.045(0.594-1.841) | 0.878 | NA |  |
| ALT |  |  |  |  |
| ≤50/>50U/L | 2.094(0.984-4.454) | 0.055 | NA |  |
| GGT |  |  |  |  |
| ≤60/>60U/L | 1.364(0.774-2.407) | 0.283 | NA |  |
| Cirrhosis |  |  |  |  |
| No/yes | 1.192(0.669-2.124) | 0.550 | NA |  |
| CNLC |  |  |  |  |
| I/II/III | 1.439(1.053-1.968) | **0.023** | 1.507(1.038-2.188) | **0.031** |
| TNM |  |  |  |  |
| I/II/III/IV | 1.272(0.936-1.728) | 0.124 | NA |  |
| Differentiation |  |  |  |  |
| I/II/III | 1.432(0.880-2.329) | 0.148 | NA |  |
| FGFR2 fusion |  |  |  |  |
| No/yes | 0.142(0.042-0.487) | **0.002** | 0.150(0.039-0.583) | **0.006** |
| PD-L1 |  |  |  |  |
| Negative/Positive | 1.451(0.802-2.624) | 0.218 | NA |  |
| CSF1R |  |  |  |  |
| Negative/Positive | 1.452(0.820-2.570) | 0.200 | NA |  |
| Tumor-infiltrating Tregs |  |  |  |  |
| Low/high | 3.043(1.249-7.415) | **0.014** | 1.512(0.588-3.888) | 0.391 |

Continued

Supplementary Table 3 Continued

| **Variables** | **Multivariate**  **HR(95%CI)** | **Univariate**  **P value** | **Multivariate**  **HR(95%CI)** | **Multivariate**  **P value** |
| --- | --- | --- | --- | --- |
| Tumor-infiltrating CD8^+^ T cells |  |  |  |  |
| Low/high | 1.347(0.764-2.375) | 0.303 | NA |  |
| Tumor-associated N1 neutrophils |  |  |  |  |
| Low/high | 0.826(0.459-1.486) | 0.523 | NA |  |
| Tumor-associated N2 neutrophils |  |  |  |  |
| Low/high | 1.382(0.732-2.611) | 0.319 | NA |  |
| Tumor-associated M1 macrophage |  |  |  |  |
| Low/high | 1.666(0.948-2.929) | 0.076 | NA |  |
| Tumor-associated M2 macrophage |  |  |  |  |
| Low/high | 1.335(0.761-2.340) | 0.313 | NA |  |

Abbreviations: CA-199, carbohydrate antigen 199; CEA, carcinoembryonic antigen; DBIL, direct bilirubin; TBIL, total bilirubin; ALP, alkaline phosphatase; AFP, alpha-fetoprotein; ALT, glutamic pyruvic transaminase; γ-GGT, gamma glutamyl transferase; CNLC, China liver cancer staging; FGFR2, fibroblast growth factor receptor 2; IQR, inter-quartile range.

**Supplementary Table 4** Antibodies used in this study

| Antibody | Company | catergory | dilution |
| --- | --- | --- | --- |
| FOXP3 | Cell Signaling Technology | 98377S | 1:500 |
| CD11b | Abcam | ab133357 | 1:1000 |
| CD206 | PTG | 60143-1-Ig | 1:1000 |
| MPO | Abcam | ab208670 | 1:1000 |
| CD80 | Abcam | Ab225674 | 1:500 |
| CD8 | Cell Signaling Technology | 70306S | 1:500 |
| CSF1R | Proteintech | 25949-1-AP | 1:500 |
| PD-L1 | Cell Signaling Technology | 13684 | 1:200 |
