# Supplementary material for: FGFR2 fusion/rearrangement is associated with favorable prognosis and immunoactivation in patients with intrahepatic cholangiocarcinoma
Source: Oncologist. 2024 Jul 10;29(12):e1734–47. doi: 10.1093/oncolo/oyae170 (PMC11630758; doi:10.1093/oncolo/oyae170)

***FGFR2* fusion/rearrangement is associated with favorable prognosis and immunoactivation in patients with intrahepatic cholangiocarcinoma**

**Figure S1 Correlation between *FGFR2* status and CSF1R/PD-L1 expression and analysis of its prognostic value.** (A-B) Representative images showing the immunohistochemistry of CSF1R and PD-L1. (C) Comparison of the IHC score of CSF1R or PD-L1 in the primary cohort samples with different *FGFR2* statuses in the primary cohort. (D, E) Kaplan‒Meier survival curve showing the OS and RFS according to CSF1R expression in the primary cohort. (F, G) Kaplan‒Meier survival curve showing the OS and RFS according to PD-L1 expression in the primary cohort. (H) Comparison of the IHC score of CSF1R or PD-L1 in the primary cohort samples with different *FGFR2* statuses in the validation cohort. (I, J) Kaplan‒Meier survival curve showing the OS and RFS according to CSF1R expression in the validation cohort. (K, L) Kaplan‒Meier survival curve showing the OS and RFS according to PD-L1 expression in the validation cohort.


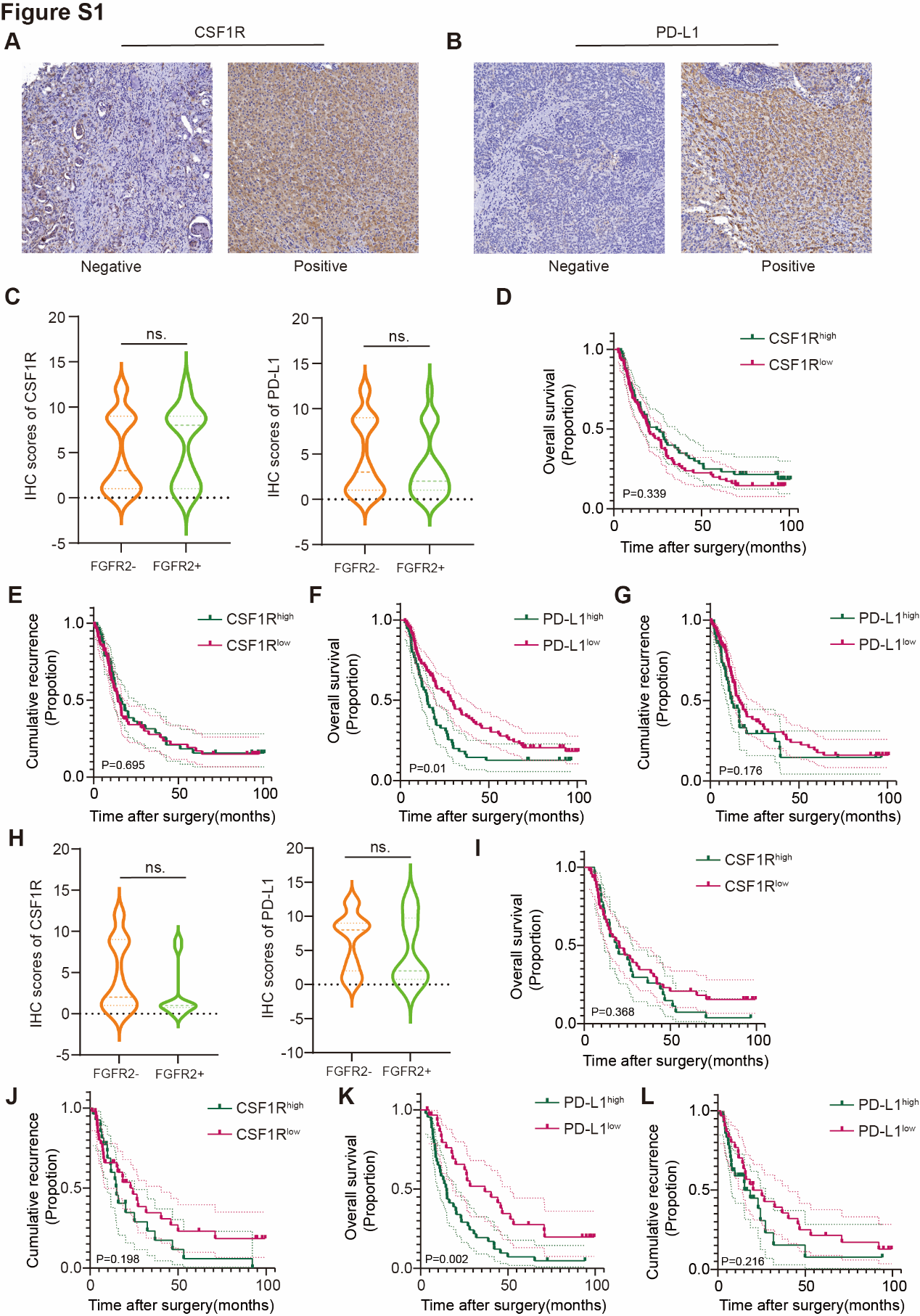


**Figure S2 Tumor-infiltrating immune cells in the validation cohort.** (A) The number of tumor-infiltrating Tregs and CD8^+^ T cells in the validation cohort. (B) Number of tumor-associated M1/M2 macrophage in the validation cohort. (C) Number of tumor-associated neutrophils in the validation cohort. (D, E) Comparison of the number of tumor-infiltrating Tregs or CD8^+^ T cells in the validation cohort samples with different *FGFR2* statuses. (F) Comparison of the number of tumor-associated neutrophils in the validation cohort samples with different *FGFR2* statuses. (G) Comparison of the number of tumor-associated neutrophils in the validation cohort samples with different *FGFR2* statuses.


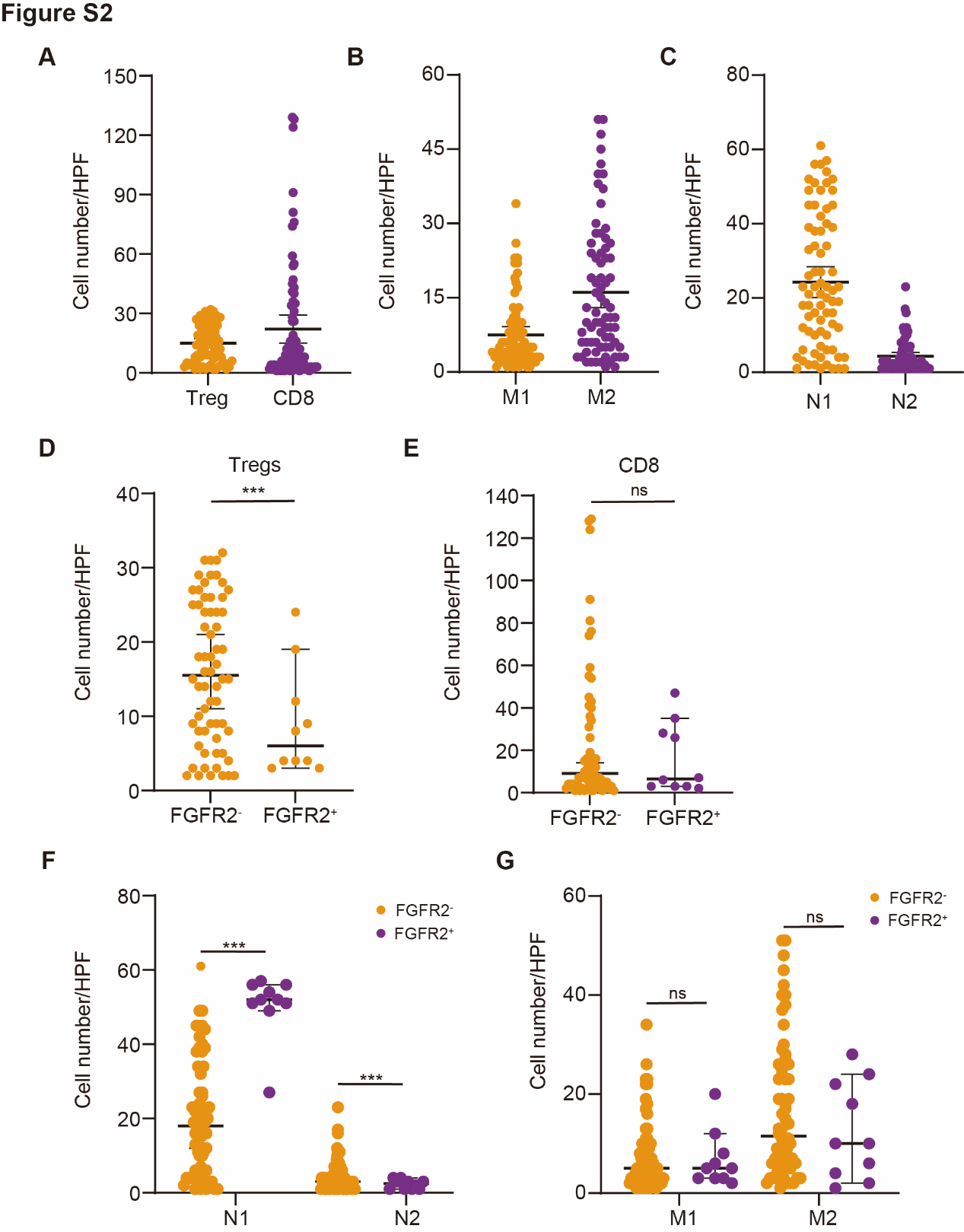


**Figure S3 Survival analysis based on the number of intratumoral infiltrating immune cells in the validation cohort.** (A, B) Kaplan‒Meier survival curve showing the OS and RFS according to number of tumor-infiltrating CD8+ T cells in the validation cohort. (C, D) Kaplan‒Meier survival curve showing the OS and RFS according to the number of tumor-infiltrating Tregs in the validation cohort. (E, F) Kaplan‒Meier survival curve showing the OS and RFS according to the number of tumor-infiltrating N1 neutrophils in the validation cohort. (G, H) Kaplan‒Meier survival curve showing the OS and RFS according to the number of tumor-infiltrating N2 neutrophils in the validation cohort. (I, J) Kaplan‒Meier survival curve showing the OS and RFS according to the number of tumor-infiltrating M1 macrophages in the validation cohort. (K, L) Kaplan‒Meier survival curve showing the OS and RFS according to number of tumor-infiltrating M2 macrophages in the validation cohort.


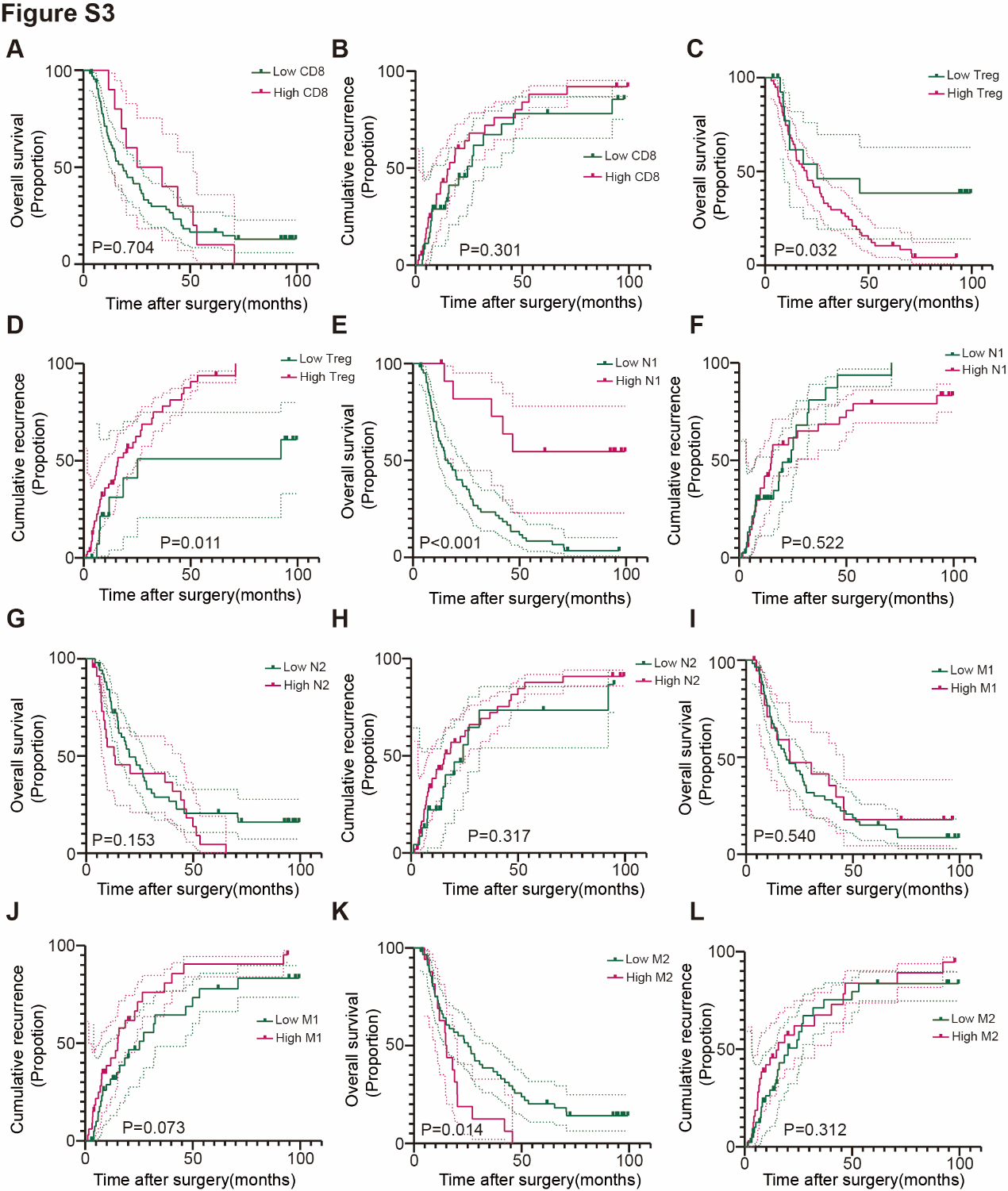

Supplement: oyae170_suppl_Supplementary_Figures [file oyae170_suppl_supplementary_figures.docx]
